# Bioprospecting of Marine Macrophytes Using MS-Based Lipidomics as a New Approach

**DOI:** 10.3390/md14030049

**Published:** 2016-03-08

**Authors:** Elisabete Maciel, Miguel Costa Leal, Ana Isabel Lillebø, Pedro Domingues, Maria Rosário Domingues, Ricardo Calado

**Affiliations:** 1 p.domingues@ua.ptmrd@ua.pt; 2 lillebo@ua.pt; 3 miguelcleal@gmail.com

**Keywords:** glycolipids, halophytes, LC-MS, lipidome, macroalgae, mass spectrometry, phospholipids, seagrasses

## Abstract

The marine environment supports a remarkable diversity of organisms which are a potential source of natural products with biological activities. These organisms include a wide variety of marine plants (from micro- to macrophytes), which have been used in the food and pharmaceutical industry. However, the biochemistry and biological activities of many of these macrophytes (namely macroalgae and halophytes, including seagrasses) are still far from being fully explored. Most popular bioactive components include polysaccharides, peptides, phenolics and fatty acids (FAs). Polar lipids (glycolipids, phospholipids and betaine lipids) are emerging as novel value-added bioactive phytochemicals, rich in *n*-3 FA, with high nutritional value and health beneficial effects for the prevention of chronic diseases. Polar lipids account various combinations of polar groups, fatty acyl chains and backbone structures. The polar lipidome of macrophytes is remarkably diverse, and its screening represents a significant analytical challenge. Modern research platforms, particularly mass spectrometry (MS)-based lipidomic approaches, have been recently used to address this challenge and are here reviewed. The application of lipidomics to address lipid composition of marine macrophytes will contribute to the stimulation of further research on this group and foster the exploration of novel applications.

## 1. Introduction

**Figure 1:**
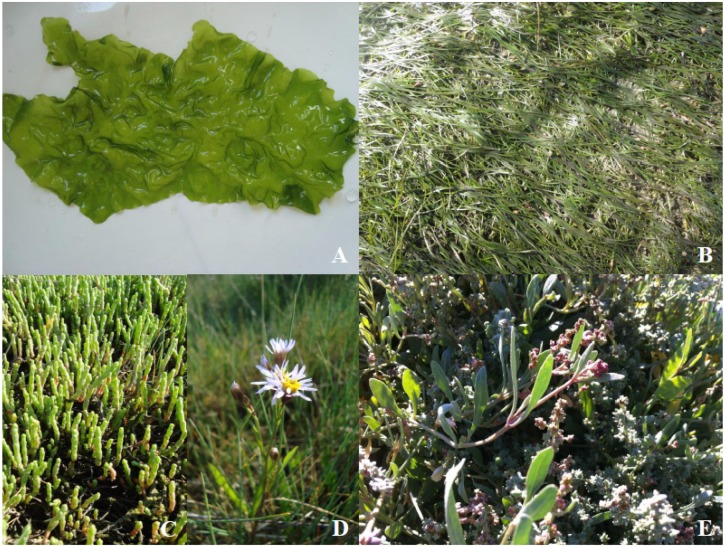
Marine macrophytes: (**A**) *Ulva lactuca* (green macroalgae); (**B**) *Zostera noltii* (seagrass); (**C**) *Salicornia ramosissima* (halophyte non-seagrass); (**D**) *Aster tripolium* (halophyte non-seagrass); and (**E**) *Halimione portulacoides* (halophyte non-seagrass). Images (**A**,**C**,**D**) by Ana I. Lillebø; (**B**) by Ana. I. Sousa; and (**E**) by Bruna Marques.

The marine environment provides a wide range of habitats that supports a remarkable biodiversity. Marine life is represented by a huge diversity of organisms with unique chemical compounds that exhibit multiple and interesting bioactivities [1], and thus hold great potential to be used as high value-added ingredients and/or as bioactive compounds. These organisms include a wide diversity of marine plants, from micro- to macrophytes. Macrophytes are represented by seaweeds (macroalgae) and halophytes (including seagrasses) (Figure 1). Halophytes can be defined as vascular plants occurring in tidal saltmarshes, mangroves and/or coastal lagoons, which are able to grow in saline environments. Marine macrophytes have long been recognized as a reservoir of potentially valuable and recoverable bioactive substances [2,3,4,5]. Indeed, this group of organisms has the potential for export markets for marine goods as natural food resources, as well as raw materials for the development of new products for industrial and health applications [2]. This potential has prompted researchers to consider them as a widely untapped source of biochemical diversity. Indeed, while the majority of new bioactive agents identified from marine macrophytes are phenolic compounds and fatty acids (FAs) [2,4], other promising molecules originating from polar lipids, including glycolipids, phospholipids, and betaine lipids, hold the potential to display antioxidant, anti-inflammatory and antimicrobial properties [6,7]. Glycolipids are important components of plants being mostly located in chloroplasts and have been demonstrated to display anti-inflammatory, antibacterial, and antiviral activity [8]. Furthermore, phospholipid molecules, known to be universal components of the lipid bilayer of cell membranes, such as phosphatidylcholine (PC), phosphatidylglycerols (PG), phosphatidylethanolamines (PE), and phosphatydylserines (PS’s), possess nutraceutical relevance. By being carriers of polyunsaturated fatty acids (PUFAs), they have the potential to be used as a valuable ingredient in functional foods, as well as in cosmetic and pharmaceutical industries.

The lipid composition of marine macrophytes can shift as an adaptive response to changes in environmental and/or physiological conditions [9]. This ability can be used to manipulate growth conditions and obtain the most desired lipid profile. While the fatty acid (FA) profile of some macrophytes has been previously described [10,11], their total lipidome is still poorly investigated. This gap of knowledge may be due to the complexity of this topic, as the lipidome comprises several distinct classes of lipids, such as triglycerides, sterols, phospholipids, glycolipids, among others. In order to truly unravel the lipidome of marine macrophytes, it is essential to employ state-of-the-art analytical methodologies that allow for the identification and quantification of several hundred lipid species. Such a task can be successfully addressed by using the most advanced mass spectrometry (MS) analytical methodologies, in an integrated lipidomic approach. Current advances in MS allow lipidomics to take the forefront in lipid analysis, as it aims to quantify the full lipidome in cells or tissues.

The present review will address the following issues: (i) new findings on lipids from marine macrophytes; (ii) new omics analytical strategies used to decipher the complex lipidome of marine macrophytes; and (iii) lipids with potential benefits for human health. The current knowledge on MS, as the main technique to identify natural products from marine macrophytes (macroalgae and halophytes, including seagrasses) will be critically discussed, pinpointing the potential of these organisms as valuable sources of health promoting biomolecules with potential medical, nutraceutical and food applications.

## 2. Marine Natural Products from Macrophytes

**Figure 2:**
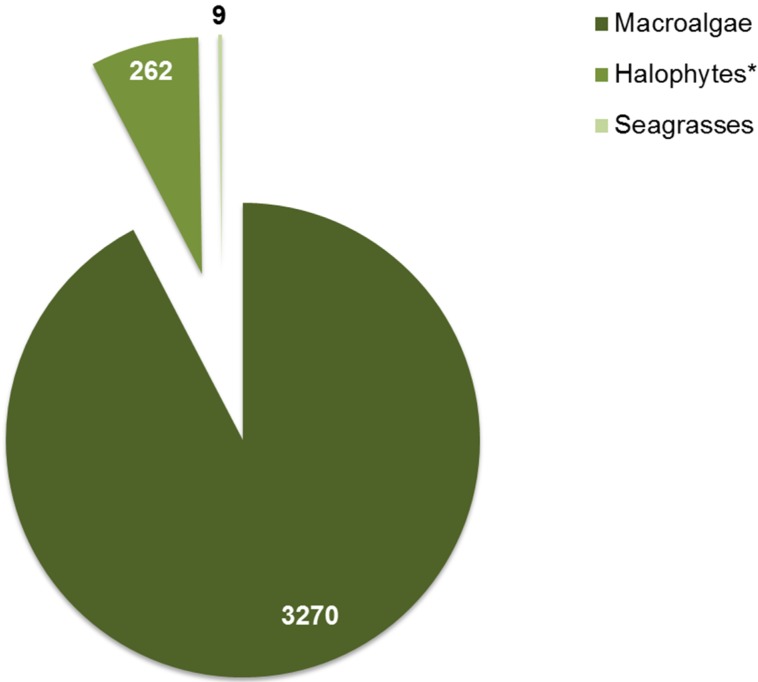
Number of marine natural products discovered from macroalgae, halophytes (* excluding seagrasses) and seagrasses between 1940 and 2014 [13].

New marine natural products (MNP) have been discovered from macrophytes, even though this group is not a bioprospecting target as popular as other marine organisms, such as invertebrates and microorganisms [12]. Nevertheless, a total of 3541 MNP have already been discovered from macrophytes between 1940 and 2014 [13]. However, these MNP are not evenly distributed among macroalgae, seagrasses and halophytes (excluding seagrasses) (Figure 2). Indeed, 92.3% of macrophytes’ MNP are associated with macroalgae, whereas halophytes (excluding seagrasses) and seagrasses solely represent 7.4% and 0.3%, respectively.

Most new MNP discovered so far have been identified from macroalgae. However, it is important to note the number of species within each group of macrophytes being addressed in the present study to better understand their chemical richness. The number of new MNP already discovered per number of species of macroalgae is approximately 7.6, whereas this ratio is 12.5 for halophytes (excluding seagrasses) and 2.3 for seagrasses. This suggests that halophytes may still have a significant bioprospecting potential that is yet to be fully unraveled. Indeed, only 21 of 605 halophyte species known to date [14] have yielded new MNP. The species *Avicennia marina* (24 MNP), *Ceriops decandra* (12 MNP), *Xylocarpus granatum* (101 MNP), *Xylocarpus moluccensis* (43 MNP) and *Xylocarpus rumphii* (11 MNP) are among the halophytes yielding most new MNP, with *Cymodocea nodosa* being the seagrass yielding the highest number of MNP to date (6 MNP). For a detailed analysis on the most bioprospected species of macroalgae, please refer to Leal *et al.* [3].

## 3. Bioactive Lipids from Marine Macrophytes

Marine macrophytes are rich in a diversified plethora of lipids. Recently, the great potential of these lipids as bioactive compounds has been demonstrated, particularly in what concerns their putative use as an anti-inflammatory, anti-proliferative, anti-microbial and anti-oxidative [4,7]. The presence of these compounds in marine macrophytes raises their biotechnological potential and their commercial value in pharmaceutical, medical, cosmetic and nutraceutical applications, as well as for food and feed.

Lipids are a large group of natural compounds which includes: fatty acids, waxes, sterols, carotenoids, mono-, di- and triacylglycerols (TGs), phospholipids (PLs), glycolipids (GLs) and betaine lipids. In the following section, we will describe the bioactive lipid classes already identified in marine macrophytes, as well as their variation according to each type of macrophyte. The present work surveyed the published scientific literature of polar lipids and fatty acids identified from macrophytes between 1971 and 2015 using the online database Web Knowledge by Thompson Reuters (available at http://apps.webofknowledge.com) and database Elsevier Scopus (available at http://www.scopus.com, consulted between October and November 2015). The following search terms, as well as their combination, were used to retrieve the information synthetized in this review: fatty acids, glycolipids, halophytes, LC-MS, macroalgae, phospholipids, polar lipids, seagrasses, and sterols).

### 3.1. Fatty Acids

FAs are one of the most simple lipid species, being composed of a carboxylic acid with long aliphatic chains. Macrophytes usually contain an even number of carbons between C4 and C28. However, the presence of FA with an unusual number of carbons has been reported in some macroalgae and halophyte species (between C15 and C21) [15,16,17]. FAs can also be classified based on the absence or presence of double bonds, as well as their number; saturated FAs (SFAs) have no double bonds, monounsaturated FAs (MUFAs) have one double bond, while PUFAs have two or more double bonds. The position of the double bonds from the methyl end also distinguishes the FA in *n*-3 (or omega-3) or *n*-6 (or omega-6), depending on whether the double bond is positioned at C3-C4 (*n*-3) or at C6-C7 (*n*-6) from the terminal of the fatty acyl chain. It is also common to find oxygenated FA such as hydroxyl, keto, epoxy and oxo, which are usually called oxylipins. These oxylipins can be formed by enzymatic oxidation of FA mediated by specific lipoxygenases and are key players in the defense response of plants [18]. FAs are usually present in marine macrophytes esterified in more complex lipids such as phospholipids, glycolipids, betaine lipids and triglycerides. Marine lipids are rich in PUFAs with *n*-3 FAs such as eicosapentaenoic acid (EPA) and docosahexaenoic acid (DHA). However, it must be highlighted that the fatty acid composition may vary with species, even within the same phyla, and is also dependent on environmental and growth conditions [19]. Marine green macroalgae (Chlorophyta), the seagrass *Zostera marina* and other halophytes are rich in C18 (α-linolenic acid (ALA), stearic acid (STA) and linoleic acid (LA)); red macroalgae (Rhodophyta) are rich in C20 PUFAs (arachidonic acid (AA) and eicosapentaenoic acid (EPA)); while in brown macroalgae (Ochrophyta) it is possible to find both C18 and C20 in higher amounts, although C16 can also be commonly found in marine macrophytes [20,21].

The variability found in the literature about the fatty acid composition of macrophytes can be explained by their ability to adapt their lipid metabolism to changing environmental conditions. The differences can be due to changes in nutritional resources, salinity stress, light stress and temperature; it is, therefore, usual to find seasonal differences in lipid composition [22,23,24,25,26]. This plasticity can be useful for biotechnological purposes, since environment manipulation can be used to increase the nutritional value of macrophytes, as it is performed for other marine species [27]. For example, it has been described that high salinity increases the content of 16:3*n*-3 and 18:3*n*-3 in *Ulva pertusa* [19] as well as PUFAs in halophytes (*Thellungiella halophile*, *Limonium bicolor* and *Suaeda salsa*) [28,29,30]. The effect of light was also studied by Floreto *et al.* [31] in three species of macroalgae (*Ulva pertusa*, *Grateloupia sparsa* and *Sargassum piluliferum*), who showed that high light intensity increases the content of SFA. Some studies targeting other macroalgae *(Undaria pinnatifida*, *Laminaria japonica*, *Fucus serratus*, *Egregia menziesii*, *Condrocanthus canaliculatus* and *Ulva lobate*) showed higher PUFA content in winter and autumn when compared to summer [23,25,32], as well as an increase on their *n*-6/*n*-3 PUFA ratio [23,26,32].

Marine macrophytes are an excellent reservoir of *n*-3 PUFAs, which have high nutritional value and can be consumed directly as a food resource or as nutraceutical and/or pharmaceutical supplement. It is already known that *n*-3 FAs are the precursors for the production of eicosanoids, such as resolvins and marsins, that are beneficial to health and have proven to be most effective in alleviating a number of health conditions (e.g., arteriosclerosis, hypertension, inflammation, immunoregulation, microbial, viral and tumor activity). It has also been suggested that the high content of PUFAs present in polar lipids (e.g., glycolipids and phospholipids) can provide an important contribution to the biological activities performed by these molecules [15,33].

### 3.2. Glycolipids

**Figure 3:**
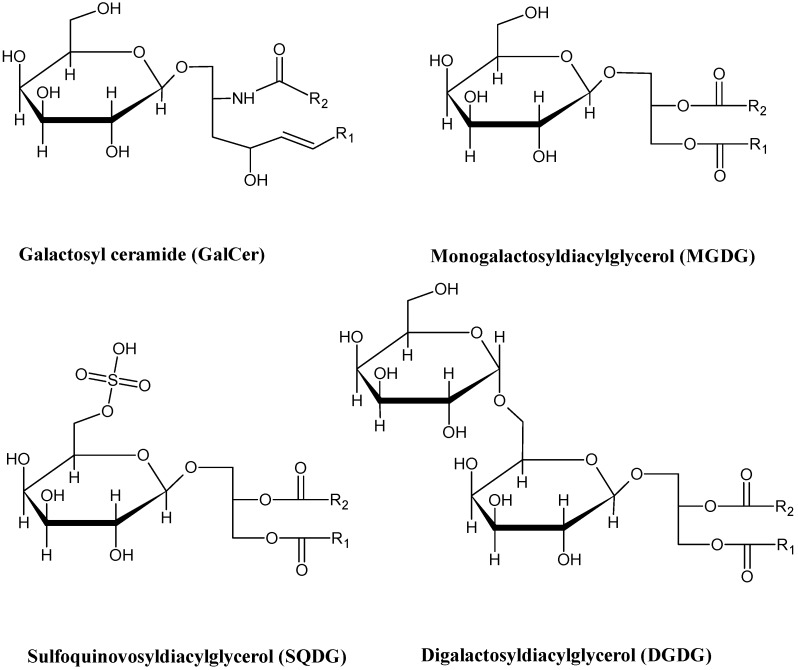
Structures of the main glycolipid classes found in marine macrophytes.

Glycolipids (GLs) constitute an important class of membrane lipids that are synthesized by prokaryotic and eukaryotic organisms. Generally, they are glycosylated derivatives of acylglycerols, termed glycoglycerolipids, and ceramide derivatives, termed glycosphingolipids. Glyceroglycolipids are the predominant GLs in marine macrophytes. Overall, marine macrophytes synthesize three major types of GL: the glycosylglycerides monogalactosyldiacylglycerides (MGDG), digalactosyldiacylglycerides (DGDG), and sulfoquinovosyldiacylglycerides (SQDG) (Figure 3).

Halophytes, as well as macroalgae, have large amounts of GLs (*ca.* 50% of total lipid content), MGDG and DGDG being greater contributors to this lipid class than SQDG (only 6%–18% of total GLs) [34,35]. Although their content varies with environmental conditions, it is possible to find higher levels of SQDG in several species, such as the halophyte *Calystegia soldanella* and several brown macroalgae, especially in high salinity environments. The DGDG/MGDG ratio increases in response to a higher saline environment in various plant groups [34]. Indeed, high DGDG/MGDG ratio and PUFAs are related to salt tolerance, as changes in this ratio may affect the structure and microviscosity of membranes and condition the resistance of organisms to environmental stress [36].

In addition, some species of red macroalgae, such as *Chondrus crispus*, *Polysiphonia lanosa*, *Ceratodictyon spongiosum* and *Halymenia* sp., contain small amounts of sphingolipids. Melo *et al.* [37] identified four molecular species of galactosylceramide (GalCer) in *Chondrus crispus* with the following fatty acid composition: 26:0/d18:1, 26:0/d18:0, 26:1/d18:1 + O and 26:0/d18:1 + O.

Most GLs contain PUFAs, especially *n*-3 FAs, the MGDG being the most unsaturated GL in halophytes, green and red macroalgae, and DGDG in brown macroalgae; SQDG is the most saturated class in all species of marine macrophytes [38]. This class of lipids has been associated with biological activities; however, it has been discussed whether FA or the polar head is responsible for their biological activities [15]. Concerning SQDGs, the presence of the sulfonate group seems to be crucial to their anti-viral activities [39] and activity against human hepatocellular carcinoma cell line (HepG2) [15].

GLs are predominantly located in photosynthetic membranes with MGDG and SQDG strictly restricted to the thylakoid membranes of the chloroplast, while DGDG is also found in extraplastidial membranes. GLs are essential to provide energy and as markers for cellular recognition because of their association with cell membranes [40]. They are also key components of membranes, protecting cells against chemical aggression from external mediums and stabilizing membrane bilayers. They play a crucial role during phosphate limitation on plants by replacing phospholipids and facilitating the survival in stressing environments. Moreover, their importance has been increasingly noted since anti-inflammatory, antitumor promoting and antiviral properties were described (Table 1). Many reports have been published about compounds isolated from macroalgae, namely, both intracellular and extracellular GLs as antitumor agents [41,42]. Recently, a great deal of interest has been expressed regarding compounds from macroalgae as potential antiviral [42,43,44] and anti-inflammatory products [41,45,46,47]. In the last two decades, the number of studies of GLs displayed by macroalgae has been increasing, as well as those on non-polar lipids and other compounds for halophytes [2,48,49]. In contrast, polar lipids in halophytes have been largely overlooked.

### 3.3. Phospholipids

**Figure 4:**
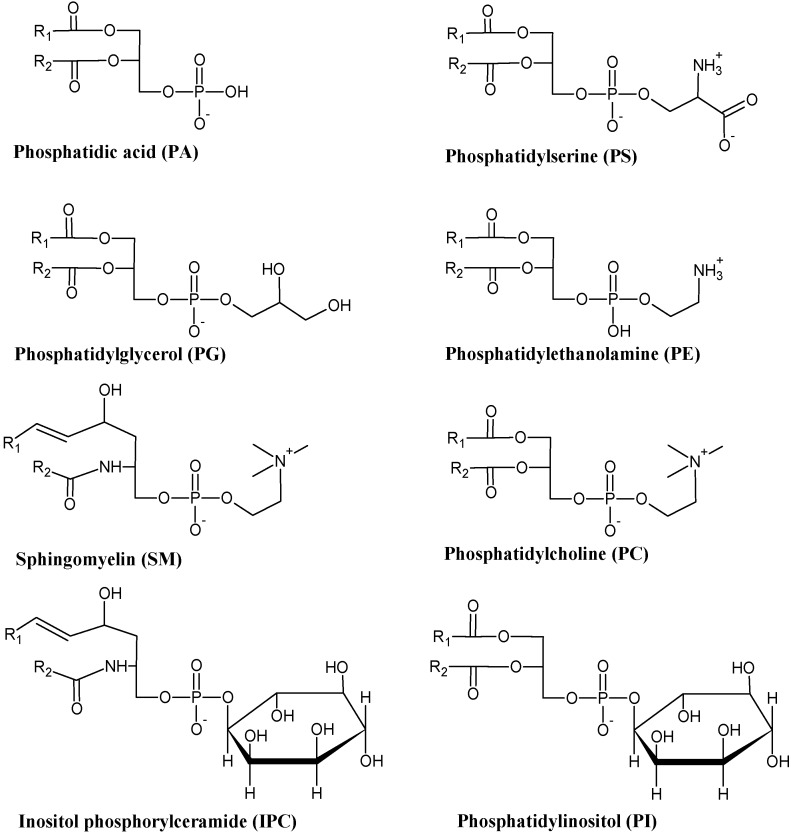
Structures of main phospholipid classes found in marine macrophytes.

Glycerophospholipids, also known as phospholipids (PLs), are polar lipids that structurally consist of a glycerol molecule linked to two FAs by an ester bond and a phosphate group that can bind a polar molecule. This portion of the PL molecule is called the head group. The composition of the head group is primordial in the classification of PLs into distinct classes. For example, if the head group is a serine, then the PL belongs to the PS class. Other classes include PC, PE, PG (Figure 4). Each class of PL includes a large number of molecular species due to the presence of different combinations of fatty acyl chains that can be linked to glycerol moiety. Overall, the phospholipid profile encompasses a great number of molecular species that can be structurally different and its quantities can vary with location and environmental conditions [22,57]. The quantity and composition of PLs is enzymatically regulated in a way that enables membranes to maintain their structure and function, in spite of their developmental stage and/or environmental variation [58].

The major PL classes in marine macrophytes are PC, PG and PE, PG being the only PL class located in significant amounts in thylakoid membranes [59]. The other PLs are located in extra-chloroplast membranes. Lipid components of living cell membranes can be adjusted to physiological and environment conditions. PC is the most abundant class of PL in halophytes, usually containing more than 70% of total PLs; for comparison purposes, one can consider that the percentage of PC often observed in higher plants is solely 45%–60% of total PLs [34]. Green macroalgae display higher amounts of PG, which range between 20% and 47% of total PLs, while in red macroalgae PC is the dominant class (60% of the total PL content) [60]. Both PC and PE classes are dominant in brown macroalgae (11.3%–29.3% of total PLs). Phosphatidic acid (PA) and phosphatidylinositol (PI) are also found in high amounts, whereas PS’s are present as a minor PL class [61,62,63]. Moreover, Vaskovsky [63] and Khotimchenko [64] also detected inositol phosphorylceramide (IPC) in red macroalgae. Both authors isolated IPC from *Gracilaria verrucosa* [65], the FA composition in its acyl chains being: myristic (9.8%), palmitic (51.7%), stearic (23.2%), oleic (9.8%) and palmitoleic acids. PLs, in contrast to GLs, contain high amounts of *n*-6 PUFA, except PGs, which contain α-linolenic acid. The major FAs in PLs are palmitic (16:0), STA (18:0), oleic (18:1), AA (20:4) and EPA (20:5). More recently, Melo *et al.* [37] also detected IPC in the red macroalgae *Chondrus crispus*, particularly fifteen molecular species of IPC with most abundant molecular species being d18:0/26:0.

Due to the dual hydrophilic and hydrophobic properties of PLs, they are mainly known for their role as building blocks for cell membranes in most organisms. In addition to their role in cellular structure and functions, they are also important for lipoproteins, which transport lipids to tissues via the blood stream. Additionally, certain PLs metabolites serve as important molecules within several signalling systems. During the last several years, more attention has been given to the beneficial health effects of PLs in animals in general and humans in particular [66].

Besides the benefit of the *n-*3 PUFAs of PLs, namely PC, they also alleviate senescence and are beneficial for cognitive functions, counter inflammatory diseases and can increase sports performance, among other beneficial properties [67]. PLs from marine macrophytes display the capacity to inhibit Hep; PLs containing *n-*3 PUFAs have more potent effects on liver and blood plasma lipid levels, compared to PLs without *n-*3 PUFAs that have been shown to increase the levels of HDL [67]. The antitumor and antiviral activities of PLs may be related to PUFAs and phosphate groups. However, the activity associated with PLs, as well as related metabolic pathways, are still not well understood.

In spite of the important roles of PLs, the full identification of their profile and their variation with external conditions are still far from being fully known. The development of modern analytical methods combining various chromatographic techniques with sensitive detection systems and MS, as well as new derivatization procedures, has led to significant progress in the deep identification of lipid profiles, as well as on the identification of new and unusual classes of lipids and FA in marine macrophytes in recent years. This knowledge is crucial to exploring the bioactive properties of polar lipids.

### 3.4. Betaine Lipids

**Figure 5:**
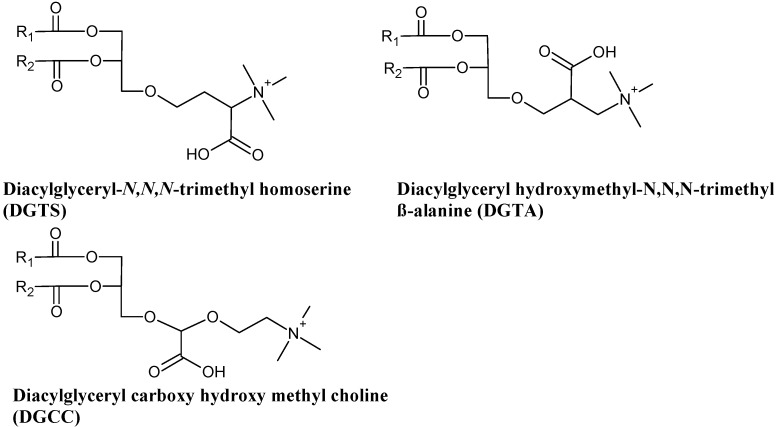
Structures of betaine lipids found in marine macrophytes.

Betaine lipids are a class of acylglycerolipids that have a quarternary amine alcohol ether-linked to a diacylglycerol moiety and lack phosphorous. They are zwitterionic at neutral pH due to their positively charged trimethylammonium group and negatively charged in carboxyl group. They can be found in lower plants and algae. Currently, three types of betaine are known to occur in macroalgae: diacylglyceryl-*N*,*N*,*N*-trimethylhomoserine (DGTS) and its structural isomer diacylglycerylhydroxymethyl-*N*,*N*,*N*-trimetyl β-alanine (DGTA) and diacylglycerylcarboxyhydroxymethylcholine (DGCC) (Figure 5).

It has been suggested that green macroalgae contain relatively higher amounts of betaine lipid DGTS (5.2%–56.5% of total polar lipids). In fact, da Costa *et al.* [68] identified not only DGTS but also monoacylglyceryl-*N*,*N*,*N*-trimethylhomoserine (MGTS) molecular species on one of these taxa (*Codium tomentosum*). The betaine lipids DGTA are minor compounds also found in thylakoid membranes of green macroalgae. In contrast, brown macroalgae contain preferentially DGTA species (7.3%–96.8% of total polar lipids) [69]. Despite their commonly being described as minor lipids in red macroalgae, Melo *et al.* [34] identified 36 DGTS molecular species in *Chondrus crispus*, the most abundant corresponding to DGTS 16:0/16:1 followed by DGTS 16:0/16:0 and 14:0/18:0.

Studies based on the analysis of fatty acid profiles showed that DGTA mainly contains saturated FAs (14:0 and 16:0) at the *sn*-1 position of the glycerol backbone and C18 unsaturated fatty acid (predominantly 18:2 and 18:3) at the *sn*-2 position; however, it can be esterified with long chain PUFAs at both the *sn*-1 and *sn*-2 positions [70,71], while DGCC contains major FAs such as palmitic, STA, oleic, AA, EPA, docosapentaenoic and DHA [72].

### 3.5. Sterols

While sterols (STs) are amphipathic compounds, not polar lipids, they are an important lipid class in marine macrophytes and are known to have already yielded a number of species with relevant bioactivity. Marine macrophytes display a large diversity of sterols, especially green macroalgae that contain chondrillasterol, poriferasterol, 28-isofucosterol, ergosterol and cholesterol, among others. Red and brown macroalgae contain one major sterol, cholesterol and fucosterol, respectively [73,74]. Sitosterol is the main sterol in halophytes [17,75].

Plant sterols, or phytosterols, can be classified based on their structure or biosynthesis, as 4-desmethyl sterols (with no substituent on carbon-4), 4α-monomethyl sterols (with one methyl group at carbon-4) and 4,4-dimethyl sterols (with two methyl groups at carbon-4). Once incorporated in the membrane bilayer, sterols regulate its fluidity and permeability. The ratio ST/PL can be used to indicate plant sensitivity to salt, with higher values helping to maintain structural integrity while decreasing the permeability of the membrane bilayer [76].

Sterols and derivatives extracted from marine macrophytes were found to play important bioactivities (e.g., anti-inflammatory and antiaterogenic). Phytosterols (C28 and C29 sterols) are important precursors of vitamin D2, cortisone and hormone flavone, playing a key role in nutraceutical industries. Phytosterol, especially β-sitosterol, has also been shown to lower total and LDL cholesterol levels in humans by inhibiting cholesterol absorption from the intestine [77]. In addition, steryl glycosides in plants and algae have also been found. The presence of glucose increase the hydrophilic part of the lipid and thus the biophysical properties of the membrane [78,79]; although the biological function are still unclear, the steryl glicoside seems to create an effective obstacle to cholesterol esterification, thus resulting in inhibition of cholesterol in the blood vessel (as reviewed in [78]). In contrast with polar lipids, sterols in halophytes have been thoroughly investigated [2].

## 4. Strategies for Lipid Analysis from Marine Macrophytes: From Extraction to Structural Characterization

Lipidomics aims to study the broad profiling of lipid molecular species that are present in living systems and, if possible, their correlation with the plethora of cellular functions mediated by lipids. Lipids are highly complex and diverse, ranging from simple structures such as FA, to more complex ones, such as PLs or GLs, which have various combinations of polar head groups, fatty acyl chains substitutions and distinct backbone structures. The full characterization of all of this structural diversity of polar lipids and their quantification is a great challenge in lipid analysis. To achieve the identification of a total lipidome, or at least to pinpoint the majority of lipids, new analytical strategies based on MS are being used. These modern approaches start with the lipid extraction from the original sample, followed by the fractionation of the total lipid extract by chromatographic methods, which can be used to obtain a rough analysis and thus analysis by MS approaches.

**Figure 6:**
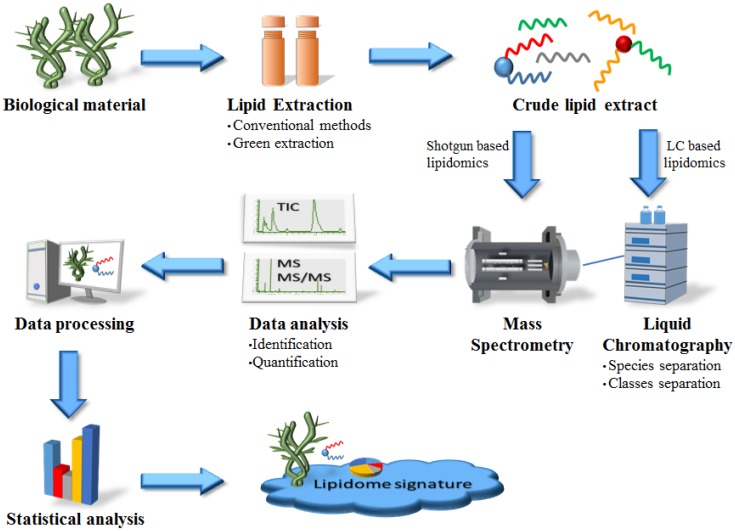
MS-based lipidomics to screen bioactive lipids from marine macrophytes.

Traditionally, lipids from marine macrophytes were analyzed by a number of chromatography methods comprising distinct analytical approaches, such as thin layer chromatography (TLC), gas chromatography (GC) and liquid chromatography (LC). All of these methods have proven to be useful for diverse purposes. TLC and LC give information about the most abundant lipid classes and GC allows for the identification of fatty acid composition. However, these methods do not provide information on all lipid classes. In order to cover the lipid profile as a whole at a molecular level, it is necessary to implement new up-to-date methodologies. MS-based methods, with or without chromatographic separation techniques, have been successfully employed in plant lipidomics [80,81], foodomics [82,83], health and disease [84], among others; due to the high resolution and sensitivity of mass spectrometers, analytical protocols are faster, less complex and require less sample manipulation. The typical lipidomics approach involves lipid extraction, separation of lipids in distinct lipid classes and lipid analysis by MS (Figure 6).

### 4.1. Methods of Lipid Extraction from Marine Macrophytes: Conventional vs. New Green Methods

Lipid analysis requires a first step of lipid extraction from selected samples. There are several experimental protocols that can be used, but they must be fast and reproducible. Moreover, they must be chosen in order to obtain the best lipid recovery. The most common methods for lipid extraction that have been applied to marine macrophytes include liquid-liquid extraction (LLE), organic solvent precipitation or solid-phase extraction (SPE) [85]. Lipid extraction protocol should also be able to extract a wide range of analytes with different polarities, with the ultimate goal of extracting the most diversified lipid structures as possible. Currently, the methods to ascertain polar lipids from marine macrophytes are mainly supported by conventional solvent extraction using organic solvents (reviewed in Section 4.1.1). Nevertheless, an effort has been made in order to develop new and eco-friendly extraction processes to obtain valuable products from natural sources and more advantages to be used for human consumption as food additives, nutraceutics or pharmaceutics products.

#### 4.1.1. Conventional Methods for Lipid Extraction

The general procedures for lipid extraction, either from animal tissues, cells or plant tissues, use organic solvents, with the most used methods differing in the type of organic solvents and the number and proportion of different organic solvents being used. Traditionally, a chloroform—methanol—water mixture is the most commonly used approach. There are two main extraction protocols using this solvent mixture: one described by Folch *et al.* in 1957 [86] and the other described by Blight and Dyer in 1959 [87]. The difference between these two methods is the proportion between chloroform (CHCl_3_) and methanol (CH_3_OH). The Folch method uses CHCl_3_/CH_3_OH (2:1), while the Blight and Dyer method uses CHCl_3_/CH_3_OH (1:2) with a subsequent addition of one volume of chloroform and one volume of water. The basic principle of these two methods is that a mixture of chloroform and methanol is initially added to the sample creating a mono-phase system that extracts the lipids from the sample matrix. Water is subsequently added to produce a biphasic system, the chloroform layer, the lower phase that contains lipids and methanol—water layer, the upper layer, with the non-lipid components. Chloroform dissolves fat, and methanol breaks down the lipid protein bonds and inactivates the lipases, while water washes the non-lipid compounds.

In some studies using Folch or Blight and Dyer methods, the chloroform was replaced by dichloromethane as a less toxic alternative [88]. Another alternative is the use of nButanol instead of chloroform, as employed in the study of the lipidome of the brown macroalgae *Sargassum thunbergii* [52]. Other solvents, such as hexane, methanol and ethanol, were tested in the lipid extraction of the halophyte *Sarcocornia ambigua* fertile shoot meal, yielding a lower efficiency in lipid extraction when compared to methanol/chloroform mixtures [10].

More recently, an extraction procedure using methyl-*tert*-butyl ether (MTBE), was introduced by Matyash *et al.* in 2008 [89]. The advantage of this method is that during phase separation the lipid-containing phase forms the upper layer, in contrast with those methods using chloroform. Furthermore, the MTBE is non-toxic and non-carcinogenic reducing health risks for exposed personnel. The MTBE method has already been applied with success to study the polar lipids of the red macroalgae *Chondrus crispus* [37].

A comparative study of different lipid extraction methods from macroalgae (*Ulva fasciata*, *Gracilaria corticata* and *Sargassum tenerrimum*) was performed by Kumari *et al.* [90]. In this work, the following extraction protocols were used: Bligh and Dyer, Folch and Cequier-Sánchez, a combination of these protocols with sonication and a buffer to improve lipid extraction was also assessed. Results showed that the macroalgal matrix, the extraction method and the buffer were paramount for lipid recoveries and should be adapted according to the desired purposes; all extraction protocols allowed for the obtaining of lipid extracts, but the buffered solvent system seemed to be more efficient for macroalgae lipid research.

#### 4.1.2. Green Extraction of Bioactive Compounds from Marine Macrophytes

New eco-friendly methods have been proposed to avoid the use of toxic solvents hazardous to health. Ultimately, eco-friendly methods should be sustainable, efficient, fast and safe, while also displaying high yields and lower costs and being easy to apply at an industrial scale. It is also important to consider that the extraction of polar lipids is sensitive and thermolabile, and that some of these molecules are found in low concentrations, thus requiring highly efficient extraction methods. The development of novel extraction methodologies may provide an alternative to the traditional methods, allowing the production of a whole range of bioactive compounds to be used as nutraceuticals and food ingredients.

Novel green extraction techniques include, among others, supercritical fluid extraction (SFE), microwave-assisted extraction, ultrasound-assisted extraction (UAE) and pressurized solvent extraction Pulsed Electric Field-Assisted Extraction and Enzyme-assisted extraction [91,92,93]. Most of these methods are based on extraction at elevated temperature and pressure, and reduced extraction time and volume of solvent. These features make them less suitable for the extraction of polar lipids (as they are sensitive to oxidation), with the exception of SFE and UAE. The advantage of SFE is the possibility of using CO_2_ instead of a solvent, thus carrying the method at low pressure and temperature. The UAE technique has the benefit of using ultrasound in solid-liquid extraction, which increases the extraction yield and promotes a faster kinetics, thus allowing the extraction of heat-sensitive compounds with minimal damage [94]. Despite the limited number of studies that investigated the applicability of these methods in marine macrophytes, it is expected that in the future they will provide higher efficiency, become time-saving, and display lower financial and environmental costs, thus becoming friendlier solutions to obtaining products from the polar lipids of marine macrophytes in a safer solvent-free environment.

### 4.2. Methods to Analyze Lipids Extracts from Marine Macrophytes

During the last several decades, the identification of polar lipids in lipid extracts was performed based on TLC, HPLC and GC. These techniques allow the screening of polar lipid classes and fatty acid profiles, as exemplified in several studies addressing the lipidome of marine macrophytes [21,30,34,38,61,63,95].

TLC allows the separation of major lipid classes, with the identification of each lipid class being based in the comparison with lipid standards applied to the same TLC plate. Some authors identified fatty acid composition within each class by running a GC analysis of FA methyl ester, obtained after methylation of the spots of each lipid class from the TLC [21,34,38,63]. Despite this strategy being widely used, its efficiency relies on the presence of a high amount of lipids and is significantly time-consuming. HPLC presents an alternative method for lipid analysis that can potentially resolve all the different lipid classes present in a crude lipid extract [96,97]. HPLC can also be coupled to several detectors, such as a light scattering detector (LSD) or a mass spectrometer. The technology of nuclear magnetic resonance (NMR) is also used in lipidomics studies, and it can be used for the structural characterization and quantification of lipid classes. However, the NMR does not provide information on the composition of the fatty acids of the individual molecular species. Usually the NMR is associated with GC-MS to achieve the detailed information of lipid species [98,99]. On the other hand, MS provides all information at the molecular species level, providing information on the chain length, degree of unsaturation and positional distribution of fatty acids at *sn*-1/*sn*-2 positions. The combination of HPLC with a mass spectrometer may result in a more detailed picture of particular lipid species within each class and of the whole lipidome in a single run [100].

#### 4.2.1. Thin Layer Chromatography (TLC)

TLC is one of the oldest techniques used for lipid separation and fractionation, still being widely used nowadays. Distinct lipid classes are usually separated by TLC using silica as the stationary phase (designated normal phase TLC). Distinct elution systems can be used depending on the polarity of the lipid classes to be isolated. For separation of nonpolar lipids (triacylglycerols, free FA, cholesterol and diacylglycerols) from more complex lipids, it is necessary to employ an elution system containing ethyl ether and hexane [101]. For the separation of polar lipid classes, namely, separation of phospholipid classes, it is necessary to use a different elution system, such as a mixture of chloroform, ethanol, water and triethylamine. In this case, the separation of polar lipid classes reflects the differences in polarity of the polar head group of polar lipids [101].

Two-dimensional TLC (2D-TLC) is also usually used for complex lipid separation. Plant lipid extract have a wide variety of lipid classes and 2D-TLC is commonly used in plant lipid analysis. The presence of glycolipids and phospholipids with similar polarities make their separation through one-dimension chromatography difficult. The combination of solvent systems for 2D-TLC is chosen based on lipid class to be isolated. Although the quality of separation is highly improved by 2D-TLC, this technique presents disadvantages compared to one-dimensional: Since only a single sample can be applied on the plate, the simultaneous application of samples and standards is not possible, thus 2D-TLC is more time-consuming [101]. An alternative to 2D-TLC is to use a multiple development in a single dimension, as applied by Olsen and Henderson [102] in lipid separation of all major algae lipid classes. Olsen and Henderson used as a first solvent system methyl acetate-isopropanol-chloroform-methanol-0.25% KCl (25:25:25:10:4, *v*/*v*/*v*/*v*); after drying, the plates were developed with the second solvent system hexane-diethylether-acetic acid (70:30:2, *v*/*v*/*v*) [102]. In these types of experiments, the high performance TLC plates are commonly used, since they have high resolution and sensitivity, presenting a good performance and reproducibility [103].

The analysis of lipids can be performed by observation of the intensity of the spots after spraying with a solution of primuline in acetone and visualization under a UV lamp, or by placing the plate in iodine vapor. After visualization of the spots, the identification is based on the comparison with migration of pure lipid standards applied to the same TLC plate. The relative quantification of lipid classes can be achieved by densitometry, based on specific colorimetric methods that can reflect the intensity of the spots or by separation of each spot and then using a specific colorimetric method. Quantification of phospholipids spots is based on phosphorous amounts, determined by Bartlett and Lewis [104]. Glycolipids can be quantified by sugar estimation using, for example, 5-methylresorcinol method or 5-hydroxy-1-tetralone, which forms a fluorescent product [105]. Analysis of the molecular species can also be achieved by scraping the spots of each lipid class, extracting the lipids in each spot with organic solvents and then analyzing the extract by MS-based approaches [106], which has the advantages of identifying all molecular species within each lipid class. The main advantage of the TLC is the possibility of obtaining a rapid screening of the sample being analyzed without the sophisticated equipment to separate lipids with different polarities. Apart from being time-consuming, other major disadvantages of this approach are its low resolution and sensitivity. TLC has been widely used to ascertain the polar lipidome of marine macrophytes, generally followed by off-line structural characterization; the majority of this characterization is performed by gas chromatography (GC) [21,26,29,45,60,61,63,65] or MS [39,52,57,107,108].

#### 4.2.2. Gas Chromatography (GC)

Methods encompassing GC are usually employed to analyze fatty acid methyl esters (FAMEs) and are typically coupled to MS (GC-MS) or flame-ionization detection (GC-FID). GC methods are sensitive to compound polarity and need derivatization steps to improve volatility. Since FAs are mainly esterified to TGs, PLs and GLs, their derivatization is performed by transmethylation in the presence of alkaline or acid catalyst, with further analysis of FAMEs [109]. BF_3_, HCl and H_2_SO_4_ are the most commonly used acid catalysts to perform acid-catalyzed transesterification; this procedure is also commonly performed in methanol, in order to generate FAMEs of FAs esterified to triacylglycerols and polar lipids [110]. Transmethylation of esterified FA can be easily performed at room temperature using methanolic KOH 2M, which is one of the most commonly used methods for FAME analysis due to its simplicity [109,111,112].

The disadvantages of using this methodology are the lower sensitivity for less abundant species and the large amount of lipids needed for derivatization. Most importantly, since GC yields information on the hydrolysis products of lipids, not on the parent compounds, the identification of classes is not complete, and the information on the fatty acid prime location is lost.

GC-FID and GC-MS have been extensively used to ascertain polar lipid composition in macroalgae and halophytes (including seagrasses), usually after the separation of polar lipid classes by TLC. Currently GC-MS-based approaches have been useful to identify the acyl composition of the polar lipidome of some macrophytes namely, *Sesuvium portulacastruma*, *Mesembryanthemum crystallinum* [113], *Suaeda altissima*, *Salicornia europaea*, *Artemisia lerchiana* [114], *Chondrus crispus*, *Ulva* sp., *Lamaria* sp., *Sargassum* sp., *Zostera* sp., among others [21,115].

#### 4.2.3. Liquid Chromatography (LC)

HPLC, also popularly known as LC, allows the fractionation of lipid extract in different lipid classes, similarly to TLC. Nowadays, the LC is usually coupled to MS (LC-MS) to promote the separation of lipid classes and its analysis in the same chromatographic run. The LC analysis of lipids can be performed using reverse phase (RPLC), normal phase (NPLC) or hydrophilic interactions (HILIC). RPLC has been most widely used in analysis of complex lipids (as reviewed in [116]). The separation is based on their hydrophobic properties; lipids are separated based on length and number of double bonds of fatty acyl composition. Thus, lipids containing longer and saturated fatty acyl chains are eluted later than those containing shorter and polyunsaturated acyl chains. On the other hand, NPLC and HILIC distinguish lipids according to their hydrophilic properties. In both cases, lipids are separated according to their polar head groups, thus being well suited when aiming to separate different lipid classes.

HILIC is an alternative to HPLC when aiming to separate polar compounds, being compatible and providing a higher sensitivity than HPLC when coupled with MS. This is especially true for electrospray, which has enhanced the popularity of coupling HILIC with MS in bioanalytical applications. HILIC coupled to MS was successfully applied to decode the lipidome of the red macroalgae *Chondrus crispus* [37] and the green macroalgae *Codium tomentosum* [68], among other red and brown macroalgae [107]. Although HILIC is increasing in popularity, RPLC is still widely used in lipidomics, namely, in plant lipidomics to separate GLs [80,117,118,119,120]. Kendal *et al.* [50] used a C18 column to show which GL species could be obtained from *Ulva armaricana* and *Solieria chordalis*, possessing anti-proliferative properties against lung tumor. RPLC was also applied in the identification of eicosanoids in the red macroalgae *Gracilaria asiatica* [121] and other oxylipins [122]. In fact, LC-MS platforms have greatly improved the resolution, sensitivity and mass range, solving problems of complex lipid separation and characterization. Due to the structural variety of polar lipids, resolving lipids in their representative classes and species relies on the combined used of MS with chromatographic methods; this approach provides the possibility of separating and concentrating on different classes, taking into consideration their physicochemical properties.

### 4.3. Mass Spectrometry-Based Lipidomics as a Valuable Tool to Find New Bioactive Lipids from Marine Macrophytes

The identification of the total lipid molecular profiles of marine plants has increased in the last several years due to the development of modern technologies, such as MS. In addition, a platform for analysis of the cellular lipidome directly from crude extracts of biological samples is becoming an attractive technique to lipid researchers. This technique allows for direct fingerprinting and quantification of hundreds of individual lipid molecular species in a single target MS or LC-MS analysis [123].

MS is a methodology widely used in lipid analysis due to its high sensitivity and capacity for identifying compounds. MS-based approaches can be either used in shotgun lipidomics or coupled to chromatographic methods. In shotgun lipidomics, lipids are identified and quantified directly from crude extracts through the direct infusion of lipids without chromatographic separation. This approach has been quite popular in the beginning of lipidomics due to its fast processing times, high reproducibility, accuracy, simplicity of operation and possibility of detecting various lipid classes in just a single run. However, it has some disadvantages that can be avoided by LC, such as ion suppression effects. The chromatographic separation prior to MS analysis can be performed either through off-line TLC or on-line HPLC. LC-MS-based methods have several advantages over off-line TLC-MS and even over direct infusion techniques. Besides reducing ion suppression effects, LC-MS allows for the identification and quantification of more than three hundred lipid species in a single run, as well as the identification and quantification of lipid molecules with the same molecular weight that can be present in different lipid classes; moreover, it is more reliable for the identification and quantification of individual molecular species, even when these are present at trace levels [37,68,124].

MS-based lipidomics analytical strategies play an important role in the identification of the lipid profile at molecular level of marine organisms. In the past several years, MS-based lipidomics has also been applied to marine macrophytes. More recently, ESI can be coupled on-line to LC prior to MS detection, which allows highly sensitive and accurate MS results, especially with more recent equipment, such as orbitrap spectrometers, that emerge as promising tools to identify biochemical signatures specific for marine macrophytes [125,126,127]. Polar lipids are often identified in MS spectra in positive and negative modes, attending to the nature of the polar head group [128]. The phospholipids, PC, lysophosphatidylcholine (LPC) and SM, as well as betaine lipids (DGTS and DGTA) are formed preferentially by positive ions, namely [M + H]^+^ ions, while PI and PG are detected through the presence of negative ions, namely [M − H]^−^ ions. Classes such as PE and PS are easily detected due to their polar group (as they display both positive and negative ions). Neutral GLs are usually identified in MS as either the ammonium adduct [M + NH_4_]^+^ or alkali metal adducts ([M + Na]^+^ or [M + Li]^+^), while SQDG are mainly detected as negative ions [M − H]^−^ (Table 2). In order to get details on the structure of lipid molecular species, it is necessary to get additional information by tandem mass spectrometry (MS/MS) studies of each ion observed in MS spectra.

Information gathered from MS/MS data and the identification of the typical fragmentation pathways of each lipid class allows several conclusions to be made about the structure of analyzed PLs or GLs, including the identification of the polar head group, the identification of the fatty acyl chains and their location at *sn-*1 *versus* *sn*-2 positions [128]. The typical MS/MS spectra of the [M + H]^+^ ions of PC, LPC and SM contain a specific product ion of the polar head at *m*/*z* 184 (H_2_PO_4_(CH_2_)_2_N^+^(CH_3_)_3_. The MS/MS spectra of the [M + Na]^+^ ions of PC and lysoPC show a neutral loss (NL) of 59 Da, due to the loss of the triethylamine from the head group (−N^+^(CH_3_)_3_), loss of 183 Da (HPO_4_(CH_2_)_2_N^+^(CH_3_)_3_) and loss of 205 Da (NaPO_4_(CH_2_)_2_N^+^(CH_3_)_3_). PC and LPC can also be analyzed in negative-ion mode as acetate adducts [M + CH_3_COO]^−^. The MS/MS spectra of these ions show a NL 74 Da, due to the loss of CH_3_COOCH_3_, corresponding to the combined loss of acetate (−59 Da) plus demethylation (−15 Da) of the choline residue and formation of the dimethylamino residue [128,129,130].

Typical MS/MS spectra of [M − H]^−^ ions of PI and lysoPI showed the specific product ion at *m*/*z* 241 a correspondent to an inositol-1,2-cyclic phosphate anion [128]. Other ions observed at *m*/*z* 223 ([C_6_H_8_PO_7_]^−^), 297 ([C_9_H_16_PO_10_]^−^) and 315 ([C_9_H_16_PO_10_]^−^) are also characteristic of PI class [128,131]. PG is also detected as [M − H]^−^ ions and their MS/MS spectra show a typical product ion at *m*/*z* 171 ([C_3_H_7_O_2_OPO_3_H]^−^) as well as the NL of 74 Da (−C_3_H_6_O_2_,) [128,132].

Although PE and lysoPE can be analyzed in both positive and negative mode, the fragmentation in positive ions is more elucidative for assigning these PL classes. The tandem mass spectra of PE [M + H]^+^ is dominated by an abundant product ion formed by the NL of 141 Da, which corresponds to elimination phosphoethanolamine head group. The MS/MS of the PE [M + Na]^+^ ions show a predominant NL of aziridine moiety from the PE polar head group (43 Da) [128]. The MS/MS of PE [M − H]^−^ ion showed the carboxylate anions R_1_COO^−^ and R_2_COO^−^ that allow for the pinpointing of the FA composition.

PS’s can form positive and negative ions; however, PS negative ions [M − H]^−^ tend to dominate. The MS/MS spectra of PS [M − H]^−^ ions yield abundant product ions that correspond to the NL of serine head group (87 Da). In the MS/MS of PS [M + H]^+^ ions, the most abundant ion results from loss of the polar head group (185 Da) [133,134].

The fragmentation under MS/MS of [M + Na]^+^ of neutral glycolipids MGDG and DGDG usually includes the loss of one hexose residue (NL of 162 Da) and, in the case of DGDG, the loss of two hexose residues (NL of 324 Da). The presence of ions at *m*/*z* 347 [Hex_2res_ + Na]^+^ and *m*/*z* 365 [Hex_2_ + Na]^+^, as well as the ion at *m*/*z* 405 corresponding to the digalactosyl glycero head group [C_15_H_26_O_11_ + Na]^+^, confirms the presence of the digalactosyl head group [44,135,136]. The typical fragmentation under MS/MS of [M − H]^−^ ions of SQDG shows the presence of ions at *m*/*z* 225 corresponding to the sulfoquinovosyl group, confirming the polar head of these polar lipids [44,135,136,137]. The typical MS/MS spectrum of [M + H]^+^ ions of the betaine lipids shows product ions at *m*/*z* 236, which is considered the diagnostic product ion of this class and corresponds to the product ion generated from cleavage of both FAs (−C_10_H_22_O_5_N^+^) [136,138]. The MS/MS spectrum of [M + Na]^+^ adducts also shows the ions at *m*/*z* 236 [135,136,139] and ions formed due to the characteristics of NL of 87 Da (−CH_2_CH_2_N^+^(CH_3_)_3_) [136,140], NL of 74 Da (−CH_2_N^+^(CH_3_)_3_) [140] and NL of 59 Da (N^+^(CH_3_)_3_) [140]. DGCC is easily detected by the dominant product ion *m*/*z* 104 [139]. The MS/MS fragmentation fingerprint data of each polar lipid class, which is summarized in Table 2, is important to define specific mass spectral detection of lipid classes. This can also be used to define specific shotgun lipidomic approaches. The presence of specific fragmentation patterns characteristic of each polar lipid class has turned precursor ion scan and neutral loss scan on powerful techniques for the identification and quantification of lipids. The multiple reaction monitoring approach, usually performed in triple quadruple spectrometers, is a target MS analysis that screens a specific parent ion/fragment ion pairs. This approach is commonly used to quantify compounds, usually using the addition of an internal standard per lipid class [141].

### 4.4. Highlights of Mass Spectrometry-Based Lipidomics in Marine Macrophytes

This section briefly highlights the applications of lipidomics conducted with LC-MS or shotgun lipidomics to study marine macrophytes, summarized in Table 3. The lipidome profile from marine macrophytes can provide a better understanding of the relation between distinct lipid species and their potential biological activity.

ESI-MS-based shotgun lipidomic analysis has been applied by Kumari *et al.* in the analysis of polar lipids to monitor lipidomic alterations at the individual lipid class level promoted by different nitrate and phosphate regimes or deprivation in *Ulva lactuca* [47]. Vu *et al.* [142] developed the direct infusion electrospray triple quadrupole MS method for studying oxylipin signatures in different stress responses in *Arabidopsis thaliana*. Nylund *et al.* [143] applied this oxylipin analysis methodology in the lipid extract from *Gracilaria* *vermiculophylla*, while Kumari *et al.* employed this approach to screen *Gracilaria dura* [144].

Ma *et al.* [145] profiled the molecular species of *Sargassum horneri* using RPLC-MS/MS. They identified 10 MGDG molecular species mainly represented by C14 and C16 saturated FAs and C18 and C16 unsaturated FA moieties. Furthermore, the authors stated that glycolipids from *S. horneri* introduced new chemical structures type of MGDGs with 18:2 that reduced the levels of TGs and FAs in adipocytes [145].

Lipidomics LC-MS approaches using HILIC coupled to MS were successfully applied to decode the lipidome of the red macroalgae *Chondrus crispus* by Melo *et al.* [37] and *Codium tomentosum* by da Costa *et al.* [68] (Table 3). Lipidomic analysis of *Chondrus crispus* allowed for the identification of 10 distinct classes of lipids and more than 180 molecular species [37]. HILIC-MS analysis of the lipid extract of *Codium tomentosum* showed the presence of over two hundred species from 12 lipid classes, which correspond to GLs (SQDG, SQMG, MGDG and DGDG), glycerophospholipids (PC, LPC, PI, PA PG and LPG) and di- and monoacyl betaine lipids. SQMG, PI and some species of monoacyl betaine lipids were also reported for the first time in green algae [68]. Ragonese *et al.* [107] used HILIC- ESI/IT-TOF-MS to analyze lipid extracts from different macroalgae and provide a reliable identification of lipid classes. *Pterocladiella capillacea* showed the most complex profile, containing several lipids classes (PG, PC, PI, LPI, PS, LPE, DGDG, SQDG, SQMG), while *Asparagopsis taxiformis* contained a single sulquinovosyl monoacylglycerol (SGMG). The lipidome of the red macroalgae *Dictyota dicotoma* was mainly represented by PLs (PC, LPE, SQMG, SQDG). This study enhances the use of the MS-base profiling of polar lipids towards the classification of marine organisms and, in general, the classification of complex lipid matrices. Although HILIC has become a relevant technique in this field, RPLC is still widely used in lipidomics, namely, in plant lipidomics to separate GLs [80,117,118,119,120]. Kendal *et al.* [50] used a C18 column to discover which glycolipid species obtained from *Ulva armaricana* and *Solieria chordalis* displayed anti-proliferative properties against lung tumor. RPLC was also applied to identify eicosanoids in the red macro algae *Gracilaria asiatica* [121] and other oxylipins [122]. In fact, LC-MS platforms have greatly improved the resolution, sensitivity and mass range, solving problems of complex lipid separation and characterization. Due to the structural variety of polar lipids, resolving lipids in their representative classes and species rely on the combined use of MS and chromatographic approaches; moreover, it also allows for the possibility of separating and concentrating different classes, taking into account their physicochemical properties [116].

## 5. Lipidomics Bioinformatics: Lipid Databases and Software

The use of lipidomics for the bioprospecting of polar lipids from macrophytes requires the use of adequate high-content databases and software tools. The information retrieved using these tools supports lipid identification and quantification. Currently, there is no universal lipid classification or dataset of compounds that can be used off-the-shelf. However, a few lipid databases are already available, such as LipidBank, LIPIDAT and LIPID MAPS, which allow researchers to start making breakthroughs in this research field. The LipidBank [146,147] classifies lipids into 17 categories, while LIPIDAT [148] database contains information mostly for phospholipids. The LIPID MAPS database classifies lipids into 8 categories: fatty acids, glycerolipids, sphingolipids, sterol lipids, prenol lipids, saccharolipids and polyketides [149]. The lipids in the LIPID MAPS Structure Database (LMSD) [150] have been sorted using this classification system and have been assigned with LIPID MAPS ID’s. At present, a total of nearly 40,000 unique lipid structures can be accessed using LMSD.

Recent analytical developments in lipidomics have focused on MS-based applications, using either direct infusion or LC-MS methods with either high- and low-resolution instruments. However, the automated analysis and interpretation of spectra remains a challenging task. The major cleavage and fragmentation pathways, as well as the mass resolution and accuracy of each ion that is detected, are often instrument-dependent. Every instrument also provides different intensity ranges for acquired spectra. Thus, spectra interpretation software is usually custom-designed for a certain mass spectrometer and/or acquisition method. In fact, there are several MS instruments, and nearly each company that commercializes them has developed its own and unique proprietary data format and software for storing and handling data. The software packages available for lipid MS data analysis, both commercial and freeware, were developed for specific types of applications and data acquisition modes. Specialized software packages such as LipidView™ (Sciex) [151] have been developed for a multiple precursor ion and neutral loss scanning. LipidSearch™ (Thermo Scientific), on the other hand, was developed jointly by academic staff and MKI (Tokyo, Japan) [152] and holds a set of tools specifically aimed for LC/MS-based lipidomics data, including high-resolution accurate-mass data generated by Orbitrap™-based mass spectrometers.

In addition to commercially available software packages, there are several free and open-source software tools and libraries that can be used for helping in MS data analysis nowadays. These software packages use open-source data formats, allowing researchers to overcome the problem of data file type incompatibility and the use of proprietary software. Among the most popular freeware package are LipidXplorer, ALEX, Lipid Blast and MS-DIAL. They are open-source software that allows for the qualitative and quantitative analysis of lipid spectra acquired using different approaches and different mass spectrometers. Both LipidXplorer [153] and ALEX [154] were designed for shotgun lipidomics using high-resolution mass spectrometers. The MS-DIAL was developed to deal with both data dependent and independent MS/MS experiments [155], while LipidBlast was developed for polar lipid analysis through MS/MS experiments and mass spectral library search, using either low- or high-resolution instruments [156].

For further processing, retrieved data are usually normalized and subjected to statistical analysis, with a number of different statistical tests. These include, MANOVA, PERMANOVA, similarity percentages (SIMPER), principal component analysis (PCA), principal coordinates analysis (PCO) and partial least-squares discriminant analysis (PLS/DA), often using different and specialized statistics packages or available freeware.

## 6. Future Perspectives

Current improvements in MS-based lipidomics open an unprecedented window of opportunity to unravel the true richness of polar lipids molecular species in marine macrophytes. It is paramount that high-throughput techniques available to screen the polar lipidome of target organisms are accompanied by reliable bioinformatics pipelines that efficiently manage generated data, so that the constraints commonly faced by other omics (e.g., metabolomics, metagenomics) can be avoided. These bioinformatics pipelines will allow a more efficient data mining and effective use of generated data, thus improving our ability to detect new MNP. Innovative applications and products will naturally emerge as we continue to gain in-depth knowledge on polar lipidomics of marine organisms (including macrophytes). Nevertheless, it is important to highlight the need to standardize protocols for the extraction and structural identification of polar lipids molecular species. The same is valid for the deposit vouchers of screened taxa, in order to allow a reliable replication of results, as well as intra- and interspecific comparisons.

## Figures and Tables

**Table 1:** Bioactivity of polar lipids in several macrophytes species.

Species Name	Lipid Class/Extract	Bioactivity	Ref.
**Green macroalgae**			
*Ulva fasciata*	SQDG	Antimicrobial (*B. subtilis* and *E. coli*)	[42]
		Antitumor (MCF-7 and HEPG2 cells)	
*Ulva armaricana*	DGDG (14:0/18:3)	Antitumor (NSCLC-N6 CELLS)	[50]
**Red macroalgae**			
*Chondria armata*	MGDG (20:5/16:0)	Antifungal (*C. albicans,*	[43]
		Antimicrobial (*Klebsiella* sp.)	
*Chondrus crispus* (cultured)		Anti-inflammatory	[45]
*Galaxoura cylindriea*	SQDG	Antimicrobial (*B. subtilis* and *E. coli*)	[42]
		Antitumor (MCF-7 and HEPG2 cells)	
*Laurencia papillose*	SQDG	Antimicrobial (*B. subtilis* and *E. coli*)	[42]
		Antitumor (MCF-7 and HEPG2 cells)	
*Osmundaria obtusiloba*	SQDG	anti-viral (HSV-1 and HSV-2)	[44]
*Palmaria palmate*	SQDG, PG	Anti-inflammatory	[46]
*Solieria chordalis*	MGDG (14:0/16:1)	Anti-tumor (NSCLC-N6 CELLS)	[50]
**Brown macroalgae**			
*Dilophys fasciola*	SQDG	Antimicrobial (*B. subtilis* and *E. coli*)	[42]
		Antitumor (MCF-7 and HEPG2 cells)	
*Fucus spiralis*	MGDG	Anti-inflammatory	[47]
*Sargassum horneri*	SQDG, DGDG	Antitumor (Caco-2 cell)	[51]
*Sargassum thumbergii*	MGDG (20:5/18:4) and (18:3/18:4)	Antifungal (*Candida albicans*)	[52]
*Sargassum wightii*	SQDG	Antimicrobial (*X. oryzae pv*.)	[53]
*Taonia atomaria*	SQDG	Antimicrobial (*B. subtilis* and *E. coli*)	[42]
		Antitumor (MCF-7 and HEPG2 cells)	
**Seagrass**			
*Cymodocea serrulata*	Chloroform extract	Antimicrobial (*P. aeruginosa* and *K. pneumoniae*)	[54]
*Halophila ovalis*	Methanolic extract	Antimicrobial (*E. coli*)	[55]
*Halophila stipulacea*	Methanolic extract	Antimicrobial (*V. cholera*)	[54]
	Chloroform extract	Antimicrobial (*S. bodii*)	
	Methanolic extract	Antimicrobial (*S. aureus)*	[55]
*Halodule pinifolia*	Methanolic extract	Antimicrobial (*S. aureus, K. pneumoniae* and *S. paratyphi)*	[54]
*Zostera capensis*	Methanolic extract	Antimicrobial (*S. paratyphi)*	[55]
	Ethyl acetate extract	Antimicrobial (*B. cereus)*	
	Ethyl acetate extract	Antimicrobial (*S. typhimurium*)	
*Zostera japonica*	Methanolic extract	Anti-inflammatory	[56]

**Table 2:** Molecular ions formed during electrospray ionization (ESI) and MS/MS fragmentation fingerprint data of each polar lipid class (in bold the most formed ion in ESI-MS).

Lipid Class	Detect Ions in MS		Precursor Ion Scan		Neutral Loss Scan	
	Negative	Positive	Negative (*m*/*z*)	Positive (*m*/*z*)	Negative (Da)	Positive (Da)
Phosphatidylcholine (PC)	[M + Ac-H]^−^	**[M + H]^+^**, [M + Na]^+^	-	184	-	-
Phosphatidylethanolamine (PE)	[M − H]^−^	**[M + H]^+^**, [M + Na]^+^	-	-	-	141
Phosphatidylglycerol (PG)	**[M − H]^−^**	[M + NH_4_]^+^, [M + Na]^+^	-	-	74	-
Phosphatidylinositol (PI)	**[M − H]^−^**	[M + NH_4_]^+^	241	-	-	-
Phosphatidylserine (PS)	**[M − H]^−^**	**[M + H]^+^**	-	-	87	185
Monogalactosyldiacylglycerol (MGDG)	[M − H]^−^	[M + NH_4_]^+^, **[M + Na]^+^**	-	243	-	179
Digalactosyldiacylglycerol (DGDG)	[M − H]^−^	[M + NH_4_]^+^, **[M + Na]^+^**	-	347	-	162
				365		341
Sulfoquinovosildiacylglycerol (SQDG)	**[M − H]^−^**	[M + NH_4_]^+^, [M + Na]^+^	225	-	-	-
Ceramide (Cer)	[M − H]^−^	**[M + H]^+^**, [M + NH_4_]^+^, [M + Na]^+^		264		
Galactosylceramide (GalCer)	[M − H]^−^	**[M + H]^+^**, [M + NH_4_]^+^, [M + Na]^+^	-	264	-	162
						180
Inositolphosphoceramide (IPC)	[M − H]^−^	**[M + H]^+^**, [M + NH_4_]^+^, [M + Na]^+^	-	223	-	162
				241		180
				259		
Diacylglyceryl-*N*,*N*,*N*-trimethylhomoserine (DGTS)		**[M + H]^+^**	-	236	-	87
						74
						59
Diacylglycerylhydroxymethyl-*N*,*N*,*N*-trimetyl β-alanine (DGTA)		**[M + H]^+^**		236		87
						74
						59
Diacylglycerylcarboxyhydroxy methylcholine (DGCC)		**[M + H]^+^**		104		

**Table 3:** Polar lipid classes in marine macrophytes analyzed by MS-based approaches.

Species Name	MS Approach	Extraction Method	Glycolipids	Phospholipids	Betaine Lipids	Ref.
**Green macroalgae**						
*Codium tomentosum*	HILIC LC-MS*^n^* ESI-LXQ-IT	CH_3_OH	SQDG (20), SQMG (4), DGDG (22), MGDG (10)	PG(22), LPG(8) PA(9), PI (13), LPC (11), PC(62)	DGTS (43), MGTS (16)	[68]
*Enteromorpha intestinalis*	HILIC-LC-MS ESI/IT-TOF	Folch	SQDG (1), SQMG (1)			[107]
*Ulva armaricana*	LC-IT-TOF	CH_3_OH:CHCl_3_ (1:1, *v*/*v*)	DGDG			[50]
*Ulva fasciata*	LC-MS*^n^* ESI-QqQ	CH_3_OH:CHCl_3_ (2:1, *v*/*v*)	SQDG (1), SQMG (1)			[42]
*Ulva lactuca*	ESI-Q-TOF-MS*^n^*	Bligh and Dyer	MGDG, DGDG, SQDG	PG, LPG, PC, LPC, PS, PA, PI	DGTS	[57]
*Ulva rigida*	HILIC-LC-MS ESI/IT-TOF	Folch	SQDG (1)	PC (4), LPE (1)		[107]
**Red macroalgae**						
*Asparagopsis taxiformis*	HILIC-LC-MS ESI/IT-TOF	Folch	SQMG			[107]
*Chondria armata*	offline TLC-ESI-QTOF-MS*^n^*	CH_3_OH + isolation in CHCl_3_	MGMG (2), MGDG (3), SQMG (2)	PG (4)		[43]
*Chondrus crispus* (cultured)	HILIC LC-MS*^n^* ESI-LXQ-IT	MTBE:CH_3_OH	DGDG (19), SQDG (14)	PG (18), LPG (2), PC (60), LPC (8), PA (14)	DGTS (14)	[37]
*Chondrus crispus* (cultured)	Off-line LC-Q-MS^n^	CH_3_OH and several fraction based on EtOAc blends	MGDG (6), DGDG (2)			[45]
*Galaxoura cylindriea*	LC-MS*^n^* ESI-QqQ	CH_3_OH:CHCl_3_ (2:1, *v*/*v*)	SQMG, SQDG			[42]
*Laurencia papillose*	LC-MS*^n^* ESI-QqQ	CH_3_OH:CHCl_3_ (2:1, *v*/*v*)	SQMG, SQDG			[42]
*Palmaria palmata*	Reverse-phase LC-Q-MS*^n^*	CH_3_OH:CHCl_3_ (1:1, *v*/*v*)	MGDG (2), DGDG (3), SQDG (2)	PG (2), PE (1)		[46]
*Pterocodiella capillacea*	HILIC-LC-MS-ESI/IT-TOF	Folch	DGDG, SQDG, SQMG	PG, PC, PI, LPI, PS, LPE		[107]
*Osmundaria obtusiloba*	Off-line API-ESI-QqQ-MS*^n^*		MGDG (1), DGDG (1), SQDG (1), SQMG (1)			[44]
*Solieria chordalis*	LC-IT-TOF	CH_3_OH:CHCl_3_ (1:1, *v*/*v*)	MGDG			[50]
**Brown macroalgae**						
*Colpomenia sinuosa*	HILIC-LC-MS ESI/IT-TOF	Folch	SQMG	PC, LPE, PI		[107]
*Cystoseyra brachicarpa*	HILIC-LC-MS ESI/IT-TOF	Folch	SQDG	PG, PC		[107]
*Dictoyota dicotoma*	HILIC-LC-MS ESI/IT-TOF	Folch	SQDG, SQMG	PC, LPE		[107]
*Dilophys fasciola*	LC-MSn ESI-QqQ	CH_3_OH:CHCl_3_ (2:1, *v*/*v*)	SQDG (1), SQMG (1)			[42]
*Fucus* sp.	ESI-LTQ-MS*^n^*	CH_3_OH + fractions solvent/solvent partitioning	MGDG (2)			[47]
*Sargassum* sp.	Reverse-phase LC-ESI-QIT-MS	EtOAc	MGDG (10)			[145]
	FAB-MS*^n^*	CH_3_OH:*n*BuOH	MGDG (2)			[52]
	offline TLC-ESI-QTOF-MS*^n^*	CH_3_OH:CHCl_3_ (1:2 and 2:1, *v*/*v*)	SQDG			[39]
*Stypocaulum scoparium*	HILIC-LC-MS ESI/IT-TOF	Folch		PG, PC, PS		[107]
*Taonia atomaria*	LC-MS*^n^* ESI-QqQ	CH_3_OH:CHCl_3_ (2:1, *v*/*v*)	SQDG, SQMG			[42]
**Halophytes**						
*Aster tripolium*	LC-TOF MS	CH_3_OH/CHCl_3_/H_2_O (65:25:4, *v*/*v*/*v*)	SQDG			[108]
*Sesuvium portulacastrum*	LC-TOF MS	CH_3_OH/CHCl_3_/H_2_O (65:25:4, *v*/*v*/*v*)	SQDG			[108]
